# *Streptococcus pneumoniae* GAPDH Is Released by Cell Lysis and Interacts with Peptidoglycan

**DOI:** 10.1371/journal.pone.0125377

**Published:** 2015-04-30

**Authors:** Rémi Terrasse, Ana Amoroso, Thierry Vernet, Anne Marie Di Guilmi

**Affiliations:** 1 Université Grenoble Alpes, Institut de Biologie Structurale (IBS), 71 Avenue des Martyrs, F-38044 Grenoble, France; 2 CNRS UMR5075, IBS, F-38044 Grenoble, France; 3 CEA, DSV, IBS, F-38044 Grenoble, France; 4 Centre for Protein Engineering, Department of Life Sciences, University of Liege, Liege, Belgium; Centers for Disease Control & Prevention, UNITED STATES

## Abstract

Release of conserved cytoplasmic proteins is widely spread among Gram-positive and Gram-negative bacteria. Because these proteins display additional functions when located at the bacterial surface, they have been qualified as moonlighting proteins. The GAPDH is a glycolytic enzyme which plays an important role in the virulence processes of pathogenic microorganisms like bacterial invasion and host immune system modulation. However, GAPDH, like other moonlighting proteins, cannot be secreted through active secretion systems since they do not contain an N-terminal predicted signal peptide. In this work, we investigated the mechanism of GAPDH export and surface retention in *Streptococcus pneumoniae*, a major human pathogen. We addressed the role of the major autolysin LytA in the delivery process of GAPDH to the cell surface. Pneumococcal lysis is abolished in the ΔlytA mutant strain or when 1% choline chloride is added in the culture media. We showed that these conditions induce a marked reduction in the amount of surface-associated GAPDH. These data suggest that the presence of GAPDH at the surface of pneumococcal cells depends on the LytA-mediated lysis of a fraction of the cell population. Moreover, we demonstrated that pneumococcal GAPDH binds to the bacterial cell wall independently of the presence of the teichoic acids component, supporting peptidoglycan as a ligand to surface GAPDH. Finally, we showed that peptidoglycan-associated GAPDH recruits C1q from human serum but does not activate the complement pathway.

## Introduction

According to a recent estimation, the human genome contains only 19 000 protein-coding genes [1] while a higher number of proteins variants are required to account for the complex regulatory proteins network that control the human organism. One way to generate the required protein function diversity is the splicing of eukaryotic gene. Alternatively, a protein may display multiple independent functions. This last concept has been termed the "moonlighting hypothesis" [2].

Moonlighting proteins were first described in eukaryotic cells [3, 4, 5]. In bacteria, the first identified moonlighting enzyme was glyceraldehyde 3-phosphate dehydrogenase (GAPDH) in group A streptococci [6]. To date, more than 300 moonlighting proteins have been reported in the three domains of life [7, 8]. In bacteria, the first moonlighting proteins were identified in pathogenic Gram-positive bacteria as putative virulence factors [9, 10] but Gram-negative and non-pathogenic or commensal bacteria also display sets of moonlighting proteins. The bacterial moonlighting proteins (90 referenced so far) are conserved enzymes which functions are related to metabolism (glycolysis and tricarboxylic acid cycle), molecular chaperonin, nucleic acid modification and other cellular processes [10].

A number of pathogenic bacteria exploit GAPDH as a virulence factor [10, 11, 12]. Bacterial GAPDH proteins display multiple functions that are not always shared among species despite high sequence conservation, up to 90% protein sequence identity. GAPDH promotes bacterial adhesion and invasion of host cells [6, 13] through interaction with plasminogen and conversion to proteolytic-active plasmin. GAPDH contributes to the evasion from the immune system [14, 15]. We recently described a novel function of GAPDH: we showed that the pneumococcal GAPDH is a ligand for C1q, a key component of the classical complement pathway of the innate immune system [16].

The Gram-positive bacteria *Streptococcus pneumoniae*, also called pneumococcus is an important human pathogen that causes respiratory tract infections like pneumonia and sinusitis but also invasive diseases such as septicemia and meningitis. This bacterium is responsible worldwide for the death of around 1 million children under 5 years of age every year [17]. A striking feature of the pneumococcus is that it uses more than 10 moonlighting proteins as virulence factors including GAPDH, like enolase, aldolase, PavA and PepO [18, 19, 20, 21, 22] while other pathogenic streptococci, like *S*. *pyogenes* and *S*. *agalactiae* only exploit two in addition to GAPDH [23, 24, 25, 26].

Moonlighting proteins are involved in the virulence process of a wide range of pathogens like *Bacillus anthracis*, *Escherichia coli*, *Staphylococcus aureus*, *Listeria monocytogenes*, Mycobacteria and almost all Streptococci ([27, 28, 29, 30, 31, 32, 10], and [11] for detailed reviews). In all cases, the biological functions are carried out at the bacterial surface, which raises the question of the mode of access to the cell surface of these cytoplasmic proteins. The absence of signal peptide sequences excludes active secretion through the Sec and accessory SecA machineries as well as through the Tat system. An alternative way is that moonlighting proteins would be released from lyzed cells prior to binding to the surface of neighbouring bacteria.

In this work, we used the pneumococcal GAPDH as a moonlighting protein prototype to test this hypothesis. Our data support an autolysin-mediated *S*. *pneumoniae* lysis process in the release of GAPDH prior to binding to the peptidoglycan.

## Results

### GAPDH release in the culture medium requires pneumococcal lysis

**Fig 1:**
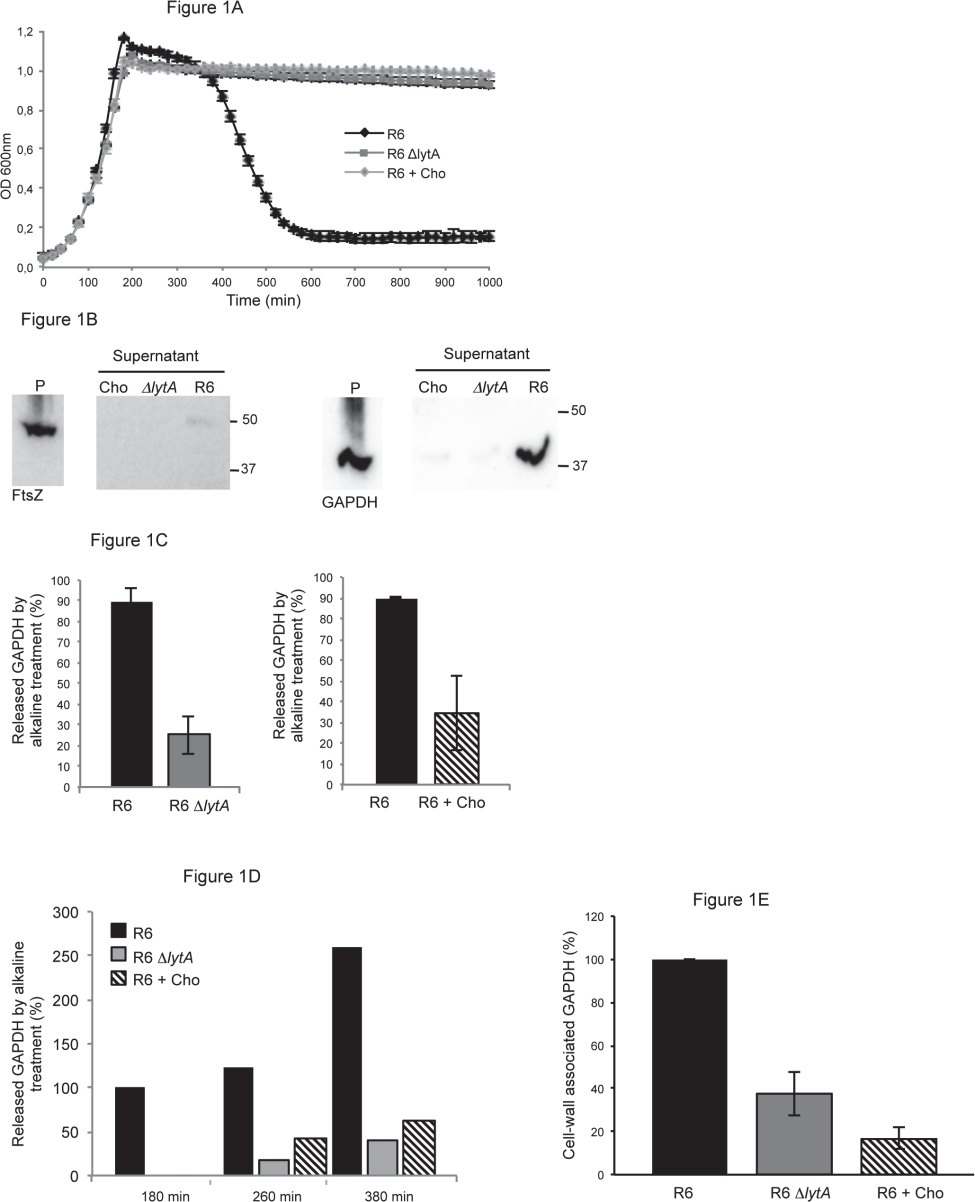
Pneumococcal lysis induced by LytA promotes GAPDH surface localization. (A) Growth profiles of the R6 strain in CY and CY + 1% Cho and of the R6 Δ*lytA* mutant in CY medium. (B) Bacterial suspensions of the R6 strain grown in CY and in CY + 1% Cho and the R6 Δ*lytA* mutant grown in CY were treated by alkaline buffer to release surface-associated proteins. Proteins present in the pellet (P) fraction corresponding to the cytoplasmic extract and in the alkaline supernatant fraction, which contains proteins detached from the cell surface, were analyzed by Western blot using appropriate polyclonal antibodies. Samples were analyzed on the same polyacrylamide gel. Left panel: detection of FtsZ (44.4 kDa) used to monitor the non-lytic effect of the alkaline treatment. Right panel: detection of GAPDH (38 kDa). Equivalent amount of loading material was determined based on OD_600nm_ values and gel scanning quantification and not on CFU measurements since the chaining morphology of the R6 Δ*lytA* mutant and the one induced by the presence of 1% Cho alters colony counting [65]. This procedure was also applied in experiments showed in Figs 1C, 1D and 1E. (C) Quantification of pneumococcal GAPDH associated to the bacterial surface in the R6 strain grown in CY and in CY + 1% Cho and in the R6 Δ*lytA* mutant grown in CY. GAPDH was detected by Western blot and quantification of the signal was performed. The average of three independent experiments is shown. (D) Same protocol as 1C. Amount of GAPDH associated to the cell wall fraction at different stages of growth. (E) Same protocol as 1C. Amount of GAPDH associated to the cell wall fraction analyzed by subcellular fractionation.

We addressed the role of pneumococcal cell lysis in the delivery process of GAPDH to the cell surface. Cell lysis is induced by hydrolytic enzymes belonging to the Choline-Binding Proteins (Cbp) family, which are bound to the phosphorylcholine (PCho) molecules associated with cell wall teichoic acids. Peptidoglycan hydrolytic enzymatic activities are harbored by LytA, LytC and CbpD [33, 34, 35, 36]. LytA behaves as the major autolysin involved in pneumococcal lysis since a *lytA* mutant strain does not display cell lysis [34] "Fig 1A". Another way to inactivate cell wall hydrolytic function is to release the Cbps from the cell surface by adding competing choline chloride in the culture medium. In these growth conditions, cell lysis is abolished to a level comparable to the one observed with the *lytA* mutant strain "Fig 1A".

The quantity of GAPDH associated to the pneumococcal surface was evaluated by alkaline elution of surface-attached proteins as described previously [16]. We checked that this procedure did not trigger cell lysis using FtsZ, an abundant cytoplasmic protein as a cell lysis marker "Fig 1B". When compared to the high quantity of cytoplasmic FtsZ "Fig 1B" (left panel, lane P), very low FtsZ was detected in the alkaline elution fraction of the R6 strain while no FtsZ was detected in the *lytA* mutant or when wild-type bacteria were grown in presence of 1% Cho "Fig 1B" (left panel, supernatant lanes). On the contrary, a large amount of GAPDH, almost equivalent to the remaining cytoplasmic quantity, is eluted from the R6 cell surface, while no protein was detected at the surface of the *lytA* mutant or in the presence of 1% Cho "Fig 1B" (right panel). These data indicate firstly that the alkaline treatment allows the release of proteins associated to the cell surface and preserves the cell integrity. Secondly, GAPDH is almost absent at the surface of cells which lysis is impaired.

To confirm the latter observation, the relative amounts of GAPDH associated to the cell surface of the R6 wild-type and *lytA* strains, and of the R6 wild-type strain grown in presence of 1% Cho were compared by Western blot and quantified. The amount of GAPDH was decreased by 70% in *lytA* mutant strain and by 65% when R6 cells were cultured in the presence of 1% Cho when compared to the wild-type strain "Fig 1C". The released level of GAPDH was analyzed at different time points during bacterial growth "Fig 1D". No GAPDH was detected at the surface of the wild-type and *lytA* mutant strains, independently on addition of Cho, at the early log phase (OD_600nm_ 0.18, 80 min, data not shown). Increasing level of GAPDH was detected at the surface of the wild-type strain from mid-log growth phase (OD_600nm_ 0.43, 180 min) to late stationary phase (OD_600nm_ 0.84, 380 min). In the context of the *lytA* mutant or when the wild-type strain is cultured in presence of Cho, the level of GAPDH associated to the cell surface was reduced by a factor 2 to 5 when compared to the wild-type strain in absence of Cho. The quantity of GAPDH associated to the cell wall was also evaluated after subcellular fractionation. As expected, the amount of GAPDH bound to the isolated cell wall was decreased in the *lytA* mutant strain and the wild-type strain grown in presence of 1% Cho by 60% and 80%, respectively, when compared to the wild-type strain grown in CY "Fig 1E".

Altogether, these data show that the presence of GAPDH at the surface of pneumococcal cells depends on the lysis of a fraction of the cell population mainly mediated by the major autolysin LytA.

### GAPDH released by cell lysis interacts with human complement factor C1q

**Fig 2:**
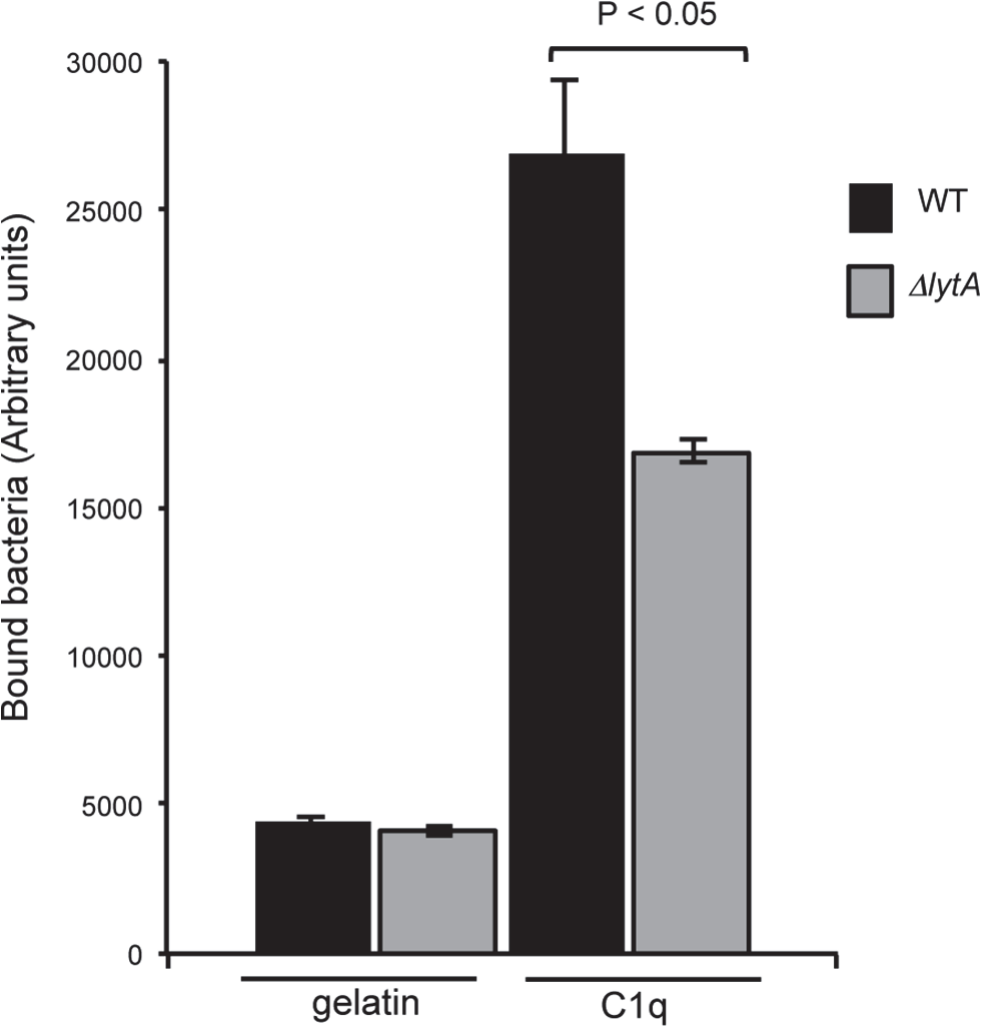
Surface GAPDH promotes binding to C1q. Bacterial culture was withdrawn at mid-exponential growth phase (OD_600nm_ 0.3). FITC-labeled bacteria were incubated for 1 h at 4°C on 1 μg of C1q coated on 96-wells plate. After five washes, the fluorescence of FITC was measured. A representative experiment of 3 independent experiments is shown including the standard deviation of triplicate points. Significance was determined by t-test analysis on 3 independent experiments.

We previously showed that GAPDH exposed at the surface of the pneumococcus interacts with the human components C1q [16]. This property was exploited to compare the level of surface GAPDH in the WT and *lytA* mutant strains. Both strains, harvested from early logarithmic growth phase (OD_600_ 0.3) and labeled with FITC were incubated with 1 μg of C1q coated on 96-wells plate. After extensive washes, the fluorescence associated to the plate was measured which correlates with the level of bacteria bound to C1q "Fig 2". The interaction of the *lytA* mutant strain with C1q is decreased by 63% when compared to the WT strain "Fig 2". This result is consistent with the lower quantity of GAPDH exposed at the surface of the *lytA* mutant and with the specific feature of GAPDH as being the unique pneumococcal ligand of C1q identified so far [16]. Altogether, the data indicate that the GAPDH released through cell lysis mediated by LytA is functional for C1q recognition.

### Interaction of GAPDH to the pneumococcal cell wall

Association of moonlighting proteins, such as GAPDH, to the bacterial cell surface suggests that the ligand might be a surface component shared among prokaryotes. We thus tested the interaction of the pneumococcal GAPDH with purified cell wall composed of peptidoglycan and teichoic acids.

**Fig 3:**
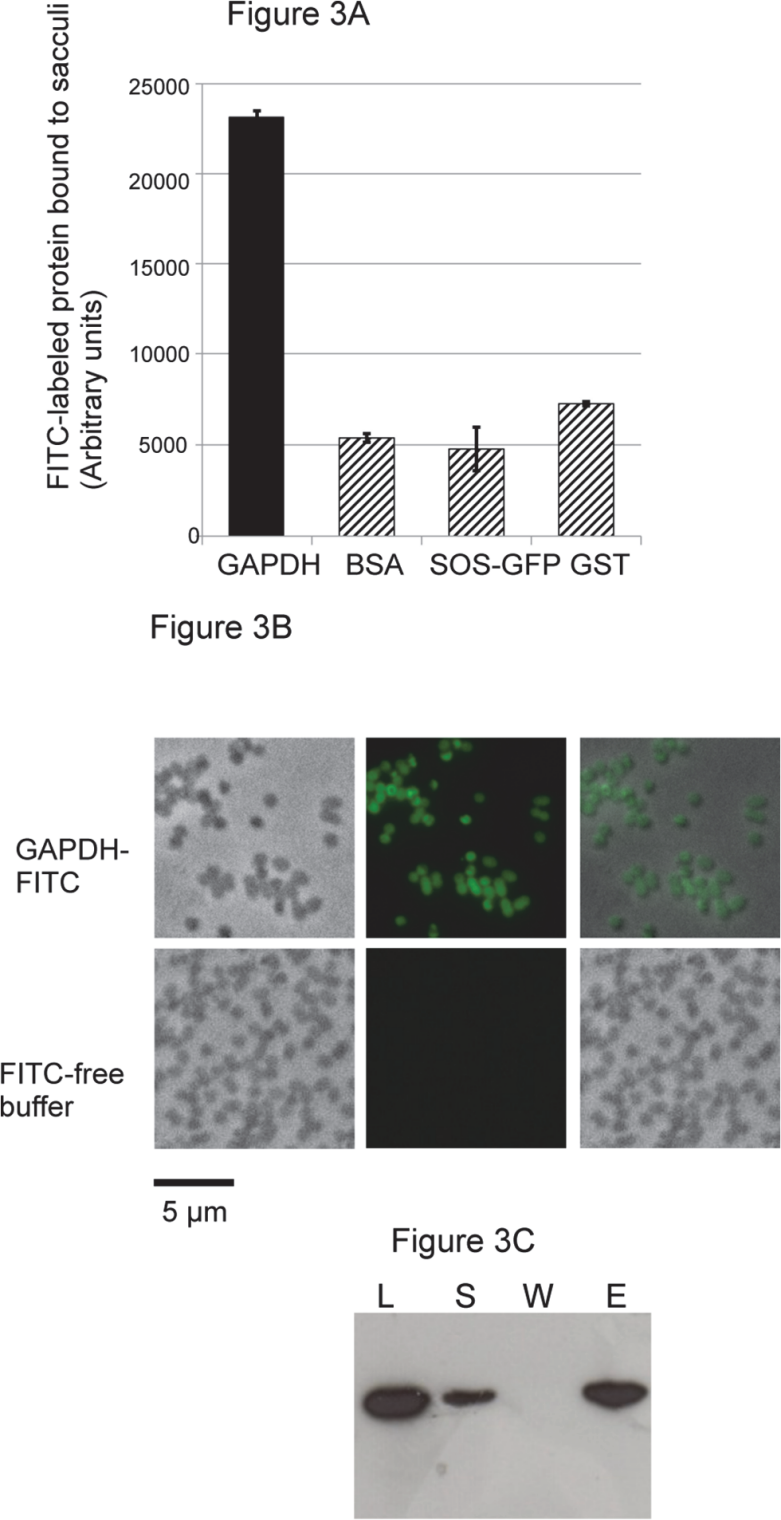
Pneumococcal GAPDH binds to the cell wall. (A) Solid-phase binding assay of FITC-labeled proteins to pneumococcal cell wall sacculi containing the peptidoglycan and the teichoic acids. (B) Microscopic images of FITC-labeled GAPDH and FITC-free buffer used a negative control bound to pneumococci cell wall sacculi. Phase contrast, fluorescence and merge pictures are shown. Scale bars, 5 μm. (C) Pull down of GAPDH with pneumococcal cell wall. The load (L) protein was mixed with the insoluble cell wall preparation. The supernatant (S) fraction containing unbound protein was recovered. After extensive wash (W), protein bound to the cell wall pellet was eluted with Laemmli buffer at 100°C for 10 min (E). The protein samples were analyzed by Western blot using an anti-His tag antibody.

We first measured the interaction of FITC-labeled GAPDH to sacculi "Fig 3A". The assay specificity was evaluated by using control proteins originating from different organisms and selected based on biochemical properties comparable to GAPDH, *i*.*e*. molecular masses and pI: pneumococcal GAPDH (38 kDa, pI 5.78), Bovine Serum Albumin (BSA, 66 kDa, pI 5.6), Glutathione-S Transferase (GST, 25.5 kDa, pI 6.09) and SOS-Green Fluorescent Protein (SOS-GFP, 30.7 kDa, pI 5.8). Control proteins were also FITC-labeled and processed similarly to the GAPDH. GAPDH displayed a four to five-fold higher level of cell wall binding when compared to the control proteins "Fig 3A". Images of GAPDH-FITC bound to pneumococcal sacculi are shown "Fig 3B". Pneumococcal sacculi, which conserve the ovococcal shape, were all decorated by GAPDH-FITC but not by free-FITC molecules "Fig 3B". Pull-down of purified recombinant GAPDH with sacculi "Fig 3C" further demonstrated the association of GAPDH to the pneumococcal cell wall.

### Pneumococcal GAPDH binds to peptidoglycan

**Fig 4:**
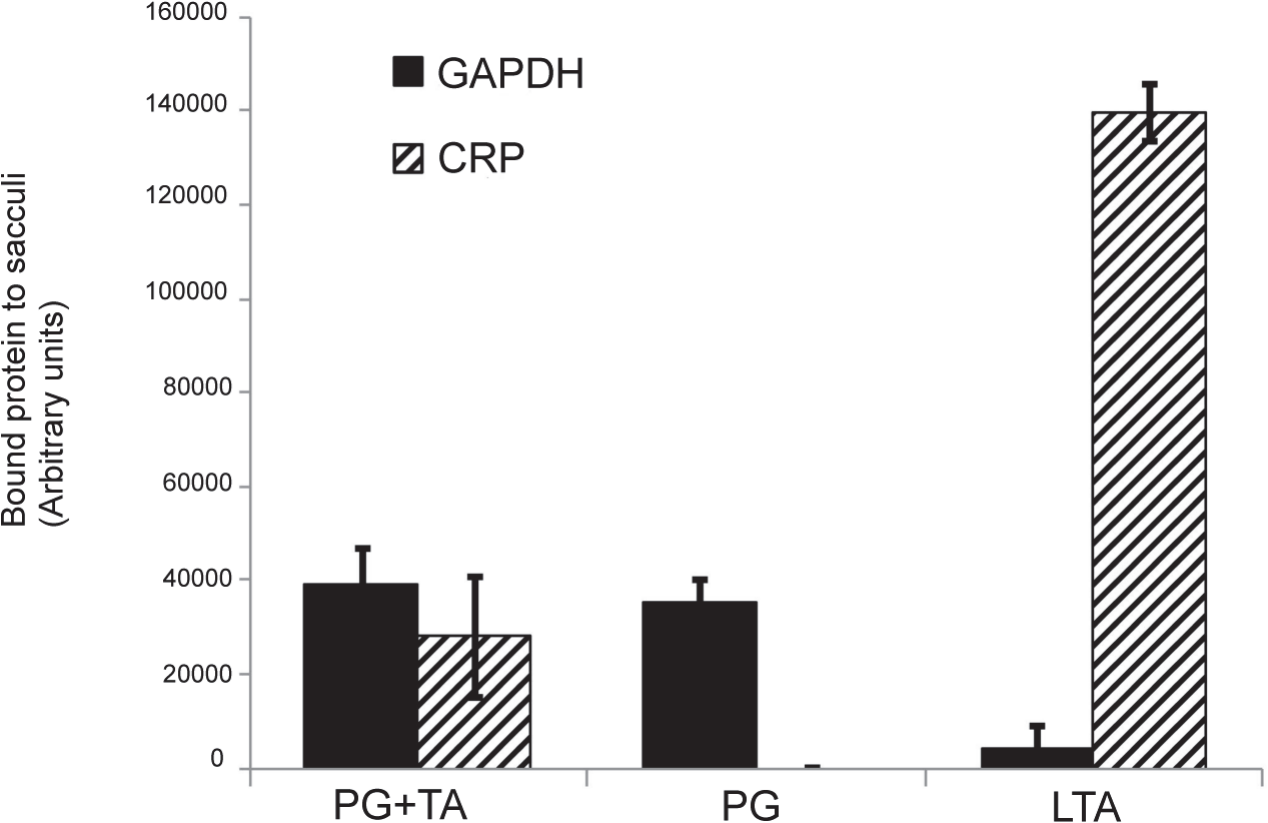
GAPDH binds to the peptidoglycan. A quantity of 100 μg of cell wall suspensions containing peptidoglycan and the teichoic acids (PG+TA) or only peptidoglycan (PG) as well as purified lipoteichoic acids (LTA) were coated on 96-well plate and incubated with GAPDH and the C-reactive protein (CRP). After extensive washes, bound proteins were immunodetected using anti-GAPDH and anti-CRP sera. Chemiluminescence was measured after extensive washes and expressed as arbitrary units. Each assay was performed three times in triplicate wells. A representative experiment is shown.

Apart from the capsule, the major polysaccharide components of the pneumococcal cell wall are the peptidoglycan (PG) and teichoic acids (TA), the latter are either membrane or peptidoglycan-anchored. Isolated pneumococcal cell wall preparations containing either the peptidoglycan and the teichoic acids or only the peptidoglycan moiety were used to investigate the respective role of both polysaccharides in GAPDH binding. As shown in Fig 4, purified GAPDH interacts with peptidoglycan independently of the presence of the teichoic acids components. Using synthetic lipoteichoic acids (LTA), no GAPDH binding could be detected. C-Reactive Protein (CRP) is a well-known protein of the innate immune system, which recognizes the PCho linked on teichoic acids moieties [37]. It was used in this experiment as an archetypal ligand for teichoic acids to ensure that the peptidoglycan cell wall preparation was indeed free from detectable traces of teichoic acids. Altogether, these data support peptidoglycan as the ligand of GAPDH at the surface of the pneumococcus.

### Peptidoglycan-associated GAPDH recruits C1q from human serum but does not activate the complement pathway

**Fig 5:**
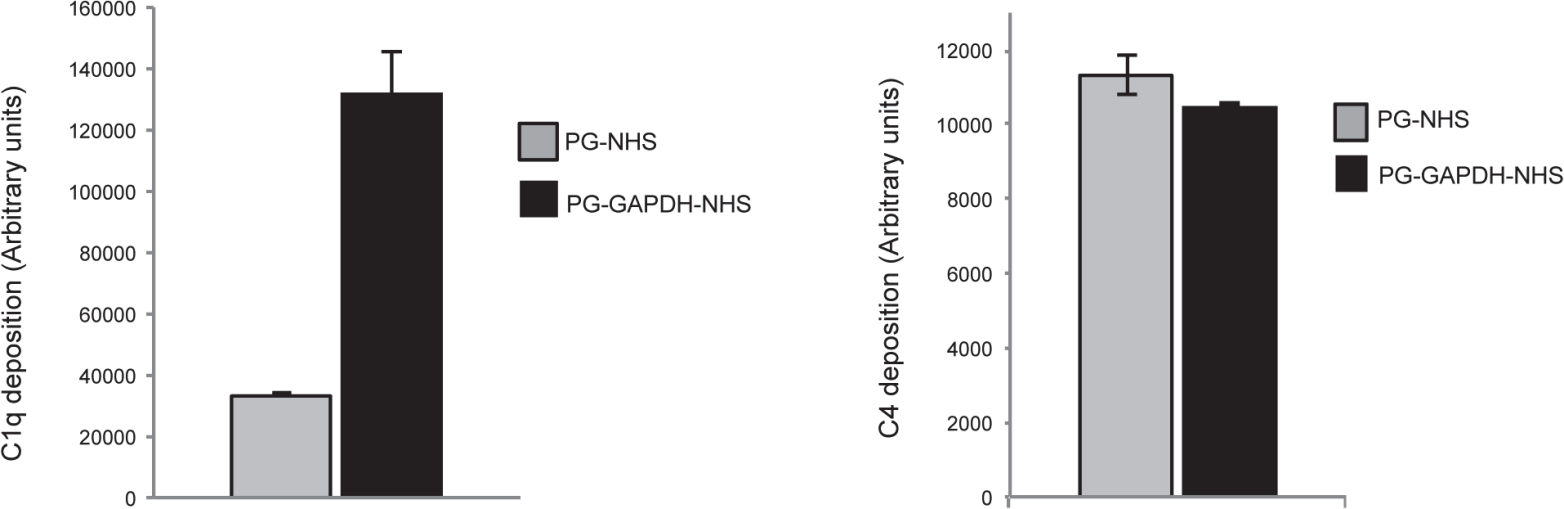
GAPDH increases C1q deposition on pneumococcal cell wall. Pneumococcal sacculi containing only peptidoglycan were deposited on 96-wells plate and incubated with or without purified GAPDH. Normal human serum (NHS) was added and subsequent C1q and C4 deposition was measured using anti-C1q and anti-C4 antibodies. A representative experiment of 2 independent experiments is shown including the standard deviation of triplicate points.

The data presented in this work indicate that GAPDH release is dependent from LytA-mediated cell lysis. Once exported, GAPDH associates to the bacterial surface through interaction with the peptidoglycan component of the cell wall. We also provide evidence that cell surface-exposed GAPDH interact with the complement factor C1q. This result is in accordance with previous work in which we also showed the ability of surface-exposed GAPDH to activate the complement pathway [16]. We sought to further investigate the role of GAPDH when associated to the bacterial cell wall. Purified GAPDH was incubated with pneumococcal sacculi only composed of peptidoglycan. Human serum was added to the complex and the levels of C1q and C4 deposited were measured "Fig 5". Peptidoglycan-associated GAPDH led to the deposition of about 5-fold more C1q than in absence of the protein, while no significant effect was observed with C4. This result suggests that peptidoglycan-bound GAPDH recruits C1q without leading to complement activation.

## Discussion

The rate of discovery of moonlighting proteins is increasing in all three domains of life as well as the importance of their roles in physiological and pathological processes [38]. Release of conserved cytoplasmic proteins is widely spread among Gram-positive and Gram-negative bacteria. Indeed, proteomic analysis have identified glycolytic enzymes, chaperonins and translation factors in culture supernatant samples of Listeria, *E*. *coli* species [39, 40] and *S*. *aureus* [41] as well as in cell wall enriched fractions of *S*. *pneumoniae* [42]. In pathogenic bacteria, moonlighting proteins are exposed at the cell surface and are mostly involved in interaction with host components. However, the mechanisms by which moonlighting proteins reach the cell surface are still unknown. Since the moonlighting proteins display crucial and pleitropic roles in virulence processes of a wide range of pathogenic microorganisms, the question of their extracellular export and their mode of attachment to the cell surface is an important issue.

Pneumococcal GAPDH, like others moonlighting proteins can not be secreted through active secretion systems since they do not contain an N-terminal predicted signal peptide. The twin-arginine translocation export pathway is absent in *S*. *pneumonia*e, which excludes the use of this system to export moonlighting proteins. The accessory secretion machinery SecA2, required for the secretion of a family of large serine-rich glycosylated proteins in Streptococci and *S*. *aureus* [43] has been shown to be involved in the secretion of cytoplasmic proteins like the superoxide dismutase A in *M*. *tuberculosis* [44] and autolytic enzyme in *L*. *monocytogenes* [45]. So far, the SecA2 pathway has not been associated to the export of GAPDH proteins. More importantly, a higher level of extracellular GAPDH was detected in *S*. *agalactiae secA2* mutant when compared to the wild-type strain [46]. In *S*. *pneumoniae*, the SecA2 pathway is present in a number of strains but absent in the D39 strain and its uncapsulated derivative strain R6. Since the latter strain was used throughout this study, the universal role of the SecA2 pathway in secretion of moonlighting proteins in the pneumococcus can be excluded. Other secretion systems present in Gram-positive bacteria like the type 4 secretion system [47] and the type 4 pili are absent in *S*. *pneumoniae* [48]. The ubiquitous YidC system is known to insert a subset of membrane proteins in the cytoplasmic membrane [49] and thus does not appear relevant to translocate soluble proteins to the cell exterior. Specific Gram-positive systems like the sortase system is not appropriate since these enzymes covalently attach proteins through a sorting motif LPxTG to nascent peptidoglycan [50]. Finally, the Esx or type 7 secretion system is absent in *S*. *pneumoniae* [51].

Cell lysis could be responsible for protein release in the extracellular medium prior to capture by the surface of live bacteria. Lysis of *S*. *agalactiae* cells mediated by detergent membrane solubilisation or cell wall synthesis inhibition through the action of beta-lactam antibiotics led to tens-fold increase of GAPDH associated to the cell surface [46]. Autolysis is a well-studied phenomenon observed when bacterial cultures reach stationary phase and is mediated by amidases, which cleave the bond between the *N*-acetylmuramic and the L-alanine residues of the peptidoglycan. The *S*. *aureus* major autolysin Atl has been shown to play a role in excretion of GAPDH since almost no protein was detected in the supernatant of *atl* mutant compared to the wild-type strain, while in a *tagO* mutant, which harbors an increased cell lysis profile, the amount of GAPDH in the supernatant was higher that in the wild-type strain [41]. We observed comparable effects in pneumococcal cells deleted from the major autolysin LytA. Higher quantity of GAPDH was detected at the surface of the wild-type strain when compared to the *lytA* mutant strain, which does not undergo lysis after the stationary phase. Similar data were obtained when cells were treated with an excess of choline chloride, which releases a family of proteins bound to the choline residues associated to the teichoic acids. LytA is a member of this Choline-Binding Proteins (Cbp) family as well as other cell wall hydrolases like CbpD and LytC [34, 35, 36]. We thus provided evidence that the presence of GAPDH at the surface of pneumococcal cells depends on the lysis of a fraction of the cell population mediated by the autolysin LytA. Beside the possibility that moonlighting proteins might be released outside the cell through cell lysis, the hypothesis that proteins might be secreted through a yet unidentified machinery should be also considered. Insertion of a hydrophobic tail at the C-terminal end of GAPDH in *S*. *pyogenes* prevented its export to the cell surface [52] Boël 2005). Deletion of a hydrophobic domain in *B*. *subtilis* enolase also affects surface exposition [53] Yang 2011). The question about a possible regulation of the cytoplasmic proteins excretion remains also open. To date, although some data suggest such process, not much is known. It was shown that automodification of *E*. *coli* enolase by covalent binding of its substrate 2-phosphoglycerate affects the enzymatic activity and was correlated to protein export [54]. A decreased active form of the *L*. *monocytogenes* superoxide dismutase was observed upon serine/threonine phosphorylation while the most active nonphosphorylated form was preferentially secreted via the SecA2 pathway [55]. More generally, bacterial glycolytic enzymes, like enolase and GAPDH are commonly detected in phosphoproteomic analysis but the physiological role of a putative post-translational modification and its role in the extracellular export have not been investigated yet [56].

We checked if the presence of GAPDH at the surface of pneumococcal cells would not be an indirect effect of protein abundance. A quantity of 2 200 molecules of GAPDH per cell of *Mycoplasma pneumoniae* had been measured [57] accounting for one of the most abundant proteins. We compared the level of cell-surface exposed GAPDH to FtsZ, a cytoplasmic protein involved in cell division, which abundance is of 3 000 molecules/cell in *S*. *pneumoniae* [58] and about 4 000 molecules/cell in *E*. *coli* [59]. Almost no FtsZ was detected at the cell surface while a large quantity corresponding to about the full cytoplasmic fraction of GAPDH was observed at the cell surface indicating that the external presence of GAPDH is not related to non-specific leakage of abundant proteins. These data are in accordance with observation in *S*. *aureus*, where the abundance of proteins in the cytoplasm and their release into the supernatant are two uncorrelated features [41].

Bergmann and Hammerschmidt were among the first to propose that moonlighting proteins could associate to the cell surface after release from the cytoplasm based on the study of pneumococcal enolase [60]. It was then shown that *Lactococcus crispatus* enolase and GAPDH bind to lipoteichoic acids [61]. Recently, the binding of *S*. *agalactiae* GAPDH to the bacterial surface was demonstrated by adding recombinant purified GAPDH to heat-inactivated and lived bacterial cells and to the surface of unrelated bacterial species, indicating that the surface ligand would be common to many bacterial species [46]. Our data bring supplemental information since we showed that the pneumococcal GAPDH binds to the peptidoglycan, the universal component of the bacterial cell wall.

Among the various roles played by bacterial GAPDH [11] one is dedicated to communication with the host immune system. GAPDH is involved in *S*. *pyogenes* evasion from neutrophil killing by inhibition of the complement-derived anaphylatoxin C5a [14]. GAPDH exposed at the surface of *S*. *agalactiae* stimulates B lymphocytes and induces an early IL-10 production that facilitates host colonization [15]. Recently, it has been shown that *S*. *agalactiae* GAPDH also acts as an inducer of murine macrophage apoptosis [46]. We identified a novel function of GAPDH in *S*. *pneumoniae* as a ligand of C1q, a soluble defense collagen, which initiates the classical complement pathway [16]. We also showed that GAPDH-C1q interaction leads to the activation of the complement pathway in terms of C4 and C3 deposition at the bacterial surface, a prerequisite step for bacterial phagocytosis by host macrophages.

The decreased level of GAPDH exposed at the surface of the *lytA* mutant correlates with a lower binding to C1q in accordance with previous results where export of GAPDH was impaired by genetic modification [16]. We wanted to investigate in further details the role of GAPDH regarding its interaction with the innate immune system. We fixed recombinant pneumococcal GAPDH to purified sacculi only composed by peptidoglycan. After incubation with human serum, C1q but not C4 was deposited to the sacculi in presence of GAPDH. This data suggests that GAPDH alone, when associated to the peptidoglycan cell wall, is able to recruit C1q but not to activate the complement pathway. It is then conceivable that the latter step would require either other effectors at the bacterial surface (teichoic acids, proteins) or a modified form (conformational change, post-translational modification) of GAPDH not available on the recombinant protein added exogenously. Alternatively, the C4 deposition detected independently of the presence of GAPDH would be triggered by the complement lectin pathway activation [62]. In conclusion, the intrinsic role of native GAPDH would be to extensively recruit and/or consume C1q to prevent further complement activation. In this context, cell-surface attached GAPDH would play a novel role in evasion of *S*. *pneumoniae* from the immune system. Although this hypothesis requires further investigation, this strategy would be different from recent findings showing that complement consumption is carried on through the interaction with C1q of a soluble moonlighting protein PepO released in the culture media [63].

## Methods

### Pneumococcal growth conditions

Pneumococcal strains were grown at 37°C with 5% CO_2_ in Todd Hewitt (TH) broth or in chemically defined C-medium supplemented with 0.1% yeast extract (CY). When indicated, 1% Choline chloride (Cho) was added to the culture media. To monitor growth curves, cells were placed into multiwell plates within a Fluostar instrument (Optima; BMG) at 37°C where the absorbance at 600 nm was recorded.

### Production of recombinant pneumococcal GAPDH

The construction of the clone encoding the pneumococcal GAPDH protein fused to a His6-tag at the N-terminus has been described previously [16]. Overnight culture of the *E*. *coli* BL21(DE3)-CodonPlus-RIL (Stratagene) strain transformed with the expression construct was used for inoculation with 500 ml of Terrific Broth medium (Euromedex) supplemented with 100 μg/ml of ampicillin and cultured at 37°C for 3 h. Protein expression was induced with 0.5mM isopropyl-β-*D*-1-thiogalactopyranoside for 16 h at 15°C. After sonication and centrifugation of the lysate (20 min, 40,000 x g), recombinant GAPDH was recovered from the soluble fraction and loaded onto a 1-ml prepacked HisTrap HP column (GE Healthcare). Column equilibration buffer was 50 mM Tris-HCl, 200 mM NaCl, 20 mM imidazole (pH 8.0). After extensive washing, His-tagged GAPDH was eluted with 60, 100, 300 and 500 mM imidazole steps in 50 mM Tris-HCl, 200 mM NaCl (pH 8.0) buffer and subsequently dialyzed against 10 mM HEPES, 150 mM NaCl, 2 mM CaCl_2_ (pH 7.4) before use for biological assays. Protein purity was checked by Coomassie Blue staining of SDS-polyacrylamide gels. Protein concentration was determined by absorbance at 280 nm.

### FITC labeling of proteins

Pneumococcal GAPDH and other control proteins were FITC-labeled using the same protocol. A volume of 40 μL of FITC (1 mg/mL) was added to 100 μL of purified protein at 6 mg/mL in 10 mM HEPES, 150 mM NaCl, 2 mM CaCl_2_ (pH 7.4). The incubation lasted for 4 h at 4°C. The protein solution was then extensively dialyzed against 10 mM HEPES, 150 mM NaCl, 2 mM CaCl_2_ (pH 7.4) to remove free FITC.

### Isolation of pneumococcal cell wall

Cell wall purification from *S*. *pneumoniae* R6 was adapted from [64]. Briefly, a 2 L culture in TH was incubated at 30°C until OD_600nm_ 0.5. Cells were harvested by centrifugation for 10 min at 4°C at 7,500 g and resuspended in 40 ml of ice-cold water. The cell suspension was poured dropwise into 40 ml of boiling 8% sodium dodecyl sulfate (SDS) and boiled for 45 min. Insoluble polymeric peptidoglycan was pelleted by centrifugation at 20°C for 20 min at 40,000 g. The pellet was washed with water until it was free of SDS. All centrifugation steps were performed at 40,000 g. The pellet was resuspended in 40 ml of 20 mM phosphate buffer, 7 mM NaCl (pH 6.9) and 200 μg/ml of α-amylase (final concentration) were added. Samples were incubated for 3 h at 20°C, with gentle shaking and then centrifuged for 60 min at room temperature (RT). The pellet was resuspended in 40 ml of 100 mM Tris (pH 8.0) and 200 μg/ml of trypsin (final concentration) was added. Samples were incubated for 18 h at 25°C with gentle shaking and then centrifuged for 60 min at RT. The pellet was resuspended in 40 ml of 100 mM Tris (pH 7.5) and 500 μg/ml of pronase was added. Samples were incubated for 3 h at 40°C and then centrifuged for 60 min at RT. The pellet was washed 3 times with cold water. At this step, samples were split and half of the pellet was treated with 49% hydrofluoridic acid during 48 h at 4°C to remove teichoic acids. Hydrofluoridic acid was removed by centrifugation and both cell wall preparations, the acid-treated and the one non treated were washed twice with 20 ml of 8 M LiCl, and then twice with 20 ml of 100 mM EDTA (pH 8.0). Cell walls were finally washed 3 times with water before being resuspended in 2 ml of water and conserved at 4°C until use.

### Elution and quantification of pneumococcal surface-exposed GAPDH

The amount of surface-exposed pneumococcal GAPDH was analyzed by an alkaline elution strategy as described previously [16]. Briefly, a 50-ml culture in late-exponential growth phase (OD_600nm_ 0.6) was harvested by centrifugation (15 min at 3,000 g). The cells were resuspended in 100 mM carbonate (pH 10) buffer and incubated for 30 min at 37°C. After centrifugation (15 min at 11,000 g), the pellet (cytoplasmic extract) and the alkaline supernatant (surface-associated fraction) were collected. Proteins were separated by SDS-PAGE, transferred on a nitrocellulose membrane and analyzed by Western blot using rabbit anti-pneumococcal GAPDH serum (1:5,000 dilution), horseradish peroxidase-conjugated anti-rabbit antibody (1:5,000 dilution, Sigma Aldrich) and ECL (Pierce) as detection reagent. The intensity of the spots was quantified using the ImageJ software. The amount of GAPDH in each sample was determined related to the GAPDH released by the wild-type strain. Pneumococcal FtsZ was detected using polyclonal rabbit serum.

### Microscopy techniques

After incubation with FITC-labeled GAPDH, cell wall sacculi suspensions were washed and deposited on microscope slides. Buffer removed after dialysis of FITC-labeled GAPDH was used as a negative control to verify that sacculi labeling would not be due to remaining free FITC molecules. Slides were observed using an Olympus BX61 optical microscope equipped with a UPFLN 100¥ O-2PH/1.3 objective and a QImaging Retiga-SRV 1394 cooled charge-coupled device camera. Image acquisition was performed using the Volocity software package and processed with Adobe Photoshop 6.0.

### Pneumococcal subcellular fractionation

The amount of pneumococcal GAPDH in the cell wall compartment of the R6 wild-type strain grown in CY or in CY supplemented with 1% Cho and of the *lytA* mutant in CY was analyzed by cell fractionation. One-tenth of a 100-ml culture in late exponential growth phase (OD_600nm_ 0.6) was centrifuged (15 min at 3,000 g), and the pellet was resuspended in 1 ml of PBS containing 100 μg/ml lysozyme and 50 U/ml mutanolysin and incubated for 2 h at 37°C. The lysate samples were submitted to SDS-PAGE and stained by Coomassie blue. The total amount of proteins in each sample was quantified using the ImageJ software in order to correct, if necessary, equivalent loads of samples. The remaining 90 ml were centrifuged (15 min, 3,000 g), the pellet was resuspended in 9 ml of PBS containing 100 μg/ml lysozyme, 50 U/ml mutanolysin, 30% sucrose and incubated for 2 h at 37°C. This lysate was centrifuged and the supernatant containing the cell wall was collected. The cell wall fractions were separated by SDS-PAGE, transferred on a nitrocellulose membrane and analyzed by Western blot using rabbit anti-pneumococcal GAPDH antibody (1:5,000 dilution), horseradish peroxidase-conjugated anti-rabbit antibody (1:5,000 dilution, Sigma Aldrich) and ECL (Pierce) as detection reagent. The intensity of the spots was quantified using the ImageJ software.

### FITC-labeled proteins binding to pneumococcal sacculi

Five mg of cell wall pneumococcal sacculi were resuspended with 50 μl of each FITC-labelled proteins at 1 mg/ml in 10 mM HEPES, 150 mM NaCl, 2 mM CaCl_2_ (pH 7.4) and incubated for 1 h at RT. Sacculi were centrifuged (5 min at 20,000 g), supernatants were removed and the pellets were resuspended in 1 mL of 10 mM HEPES, 150 mM NaCl, 2 mM CaCl_2_ (pH 7.4). This washing step was repeated five times. The sacculi pellets were finally resuspended in 50 μL of 10 mM HEPES, 150 mM NaCl, 2 mM CaCl_2_ (pH 7.4) and transferred into a black 96-well microtiter plate (Greiner Bio One). Bound proteins to pneumococcal sacculi were detected by fluorescence measurements (Fluostar Optima; BMG). Each experiment was performed in duplicate.

### Cell wall pull-down binding assay

GAPDH (10 μg/ml) was incubated with 5 mg of purified pneumococcal cell wall containing both PG and TA components in 50 μl of 10 mM HEPES, 150 mM NaCl, 2 mM CaCl_2_ (pH 7.4) for 16 h at 4°C. After centrifugation (5 min at 5,000 g), the supernatant was removed and the cell wall pellet was washed three times and resuspended in 50 μl Laemmli buffer. After incubation at 100°C for 10 min and a centrifugation step (3 min at 5,000 g), the supernatant fraction containing the eluted cell wall-bound protein was recovered. Load, supernatant, wash and bound fractions were analyzed by Western blotting using HRP-conjugated anti-His tag monoclonal antibody diluted 1:10 000 before detection with a chemiluminescent substrate (ECL; Thermo Scientific Pierce).

### Bacterial binding assay to C1q

Solid-phase binding assays were performed to measure binding of the R6 wild-type and *lytA* mutant strains to C1q. Black 96-well microtiter plates (Greiner Bio One) were coated in triplicates with 1 μg of C1q or gelatin in PBS at 4°C overnight. Saturation was performed by adding 200 μl/well of 0.2% gelatin in 10mM HEPES, 150mM NaCl, 2mM CaCl2 (pH 7.4) (HBS-C) for 1 h at RT. Five washes were performed using 200 μl of HBS-C. 100 μl of FITC-labeled bacteria (7,5.10^6^ CFU) were added to each well, and the mixture was incubated for 1 h at RT. Five washes were performed using 200 μl of HBS-C. The bacteria-associated fluorescence was measured using a multiwell fluorescence reader (Fluostar Optima, BMG Labtech).

### Complement deposition on pneumococcal sacculi and on pneumococci cells

White 96-well microtiter plates (Greiner Bio One) were coated in triplicates with 100 μg of purified pneumococcal sacculi containing only the peptidoglycan (PG) in TBS-C (50 mM Tris pH 8.0, 100 mM NaCl, 2 mM CaCl2). Saturation was performed by adding 200 μl of TBS-C-0.2% gelatin per well for 1 h at RT. Three washes were performed using 200 μl of TBS-C. Whenever required, 100 μl of GAPDH at 10 μg/ml in TBS-C was added, and the mixture was incubated for 1 h at RT. Normal Human Serum (NHS) diluted 1:25 in TBS-C was added and incubated for 1 h at RT. Three washes were performed using 200 μl of TBS-C. Bound C1q and C4 proteins were detected by adding 100 μl of rabbit anti-C1q (in house production) and rabbit anti-C4 (Abcam ab48612) antibodies (1:1,000 dilution) in TBS-C-0.02% gelatin for 1 h at RT. Following washes, secondary antibodies coupled to horseradish peroxidase (1:1,000 dilution) in TBS-C-0.02% gelatin were incubated for 1 h at RT. After three washes with TBS-C, ECL solution (Pierce) (100 μl) was added to each well and chemiluminescence was measured using a multiwell luminescence reader (Fluostar Optima; BMG).

### Solid-phase binding assay on pneumococcal sacculi

Purified pneumococcal sacculi containing the peptidoglycan (PG) and the teichoic acids (TA) components, or only the PG were used. Purified LTA from *S*. *pneumoniae* was purchased from Statens Serum Institute (Denmark) and human CRP from Calbiochem (236608). White 96-well microtiter plates (Greiner Bio One) were coated in triplicates with 100 μg of the different polysaccharide reagents. Saturation was performed by adding 200 μl of PBS-0.2% gelatin per well for 1 h at RT. Three washes were performed using 200 μl of PBS. A volume of 100 μl of GAPDH and CRP at 10 μg/ml in PBS was added to each well, and the mixture was incubated for 2 h at RT. Three washes were performed using 200 μl of PBS-0.2% Tween 20. Bound proteins were detected by adding 100 μl of mouse anti-His tag and goat anti-CRP antibodies coupled to horseradish peroxidase (1:1,000 dilution) in PBS-0.02% Tween 20–0.02% gelatin for 2 h at RT. After three washes with PBS-0.2% Tween-20, ECL solution (Pierce) (100 μl) was added to each well and chemiluminescence was measured using a multiwell luminescence reader (Fluostar Optima; BMG).
